# A novel strategy for NQO1 (NAD(P)H:quinone oxidoreductase, EC 1.6.99.2) mediated therapy of bladder cancer based on the pharmacological properties of EO9

**DOI:** 10.1054/bjoc.2001.2056

**Published:** 2001-10

**Authors:** G A Choudry, P A Hamilton Stewart, J A Double, M R L Krul, B Naylor, G M Flannigan, T K Shah, J E Brown, R M Phillips

**Affiliations:** Cancer Research Unit, Department of Pharmaceutical Chemistry, University of Bradford, Bradford, BD7 1DP; Departments of Urology, Histopathology, Bradford NHS Trust, Bradford, BD9 6RJ; Department of Medical & Scientific Affairs, NDDO Oncology, Amsterdam

**Keywords:** bioreductive drugs: EO9, mitomycin C: bladder cancer, NQO1

## Abstract

The indolequinone EO9 demonstrated good preclinical activity but failed to show clinical efficacy against a range of tumours following intravenous drug administration. A significant factor in EO9's failure in the clinic has been attributed to its rapid pharmacokinetic elimination resulting in poor drug delivery to tumours. Intravesical administration of EO9 would circumvent the problem of drug delivery to tumours and the principal objective of this study is to determine whether or not bladder tumours have elevated levels of the enzyme NQO1 (NAD(P)H:quinone oxidoreductase) which plays a key role in activating EO9 under aerobic conditions. Elevated NQO1 levels in human bladder tumour tissue exist in a subset of patients as measured by both immunohistochemical and enzymatic assays. In a panel of human tumour cell lines, EO9 is selectively toxic towards NQO1 rich cell lines under aerobic conditions and potency can be enhanced by reducing extracellular pH. These studies suggest that a subset of bladder cancer patients exist whose tumours possess the appropriate biochemical machinery required to activate EO9. Administration of EO9 in an acidic vehicle could be employed to reduce possible systemic toxicity as any drug absorbed into the blood stream would become relatively inactive due to an increase in pH. © 2001 Cancer Research Campaign   http://www.bjcancer.com


*British Journal of Cancer* (2001) **85**(8), 1137–1146
© 2001 Cancer Research Campaign
doi: 10.1054/ bjoc.2001.2056, available online at http://www.idealibrary.com on IDEAL

http://www.bjcancer.com

### A novel strategy for NQO1 (NAD(P)H:quinone oxidoreductase, EC 1.6.99.2) mediated therapy of bladder cancer based on the pharmacological properties of EO9

**GA Choudry[1,2,3], PA Hamilton Stewart[3], JA Double[1], MRL Krul[5], B Naylor[4], GM Flannigan[3], TK Shah[3], JE Brown[2] and RM Phillips[1]**

[1]Cancer Research Unit and [2]Department of Pharmaceutical Chemistry, University of Bradford, Bradford BD7 1DP; [3]Departments of Urology and [4]Histopathology, Bradford NHS Trust, Bradford BD9 6RJ; [5]Department of Medical & Scientific Affairs, NDDO Oncology, Amsterdam

**Summary** The indolequinone EO9 demonstrated good preclinical activity but failed to show clinical efficacy against a range of tumours following intravenous drug administration. A significant factor in EO9's failure in the clinic has been attributed to its rapid pharmacokinetic elimination resulting in poor drug delivery to tumours. Intravesical administration of EO9 would circumvent the problem of drug delivery to tumours and the principal objective of this study is to determine whether or not bladder tumours have elevated levels of the enzyme NQO1 (NAD(P)H:quinone oxidoreductase) which plays a key role in activating EO9 under aerobic conditions. Elevated NQO1 levels in human bladder tumour tissue exist in a subset of patients as measured by both immunohistochemical and enzymatic assays. In a panel of human tumour cell lines, EO9 is selectively toxic towards NQO1 rich cell lines under aerobic conditions and potency can be enhanced by reducing extracellular pH. These studies suggest that a subset of bladder cancer patients exist whose tumours possess the appropriate biochemical machinery required to activate EO9. Administration of EO9 in an acidic vehicle could be employed to reduce possible systemic toxicity as any drug absorbed into the blood stream would become relatively inactive due to an increase in pH. © 2001 Cancer Research Campaign http://www.bjcancer.com

**Keywords**: bioreductive drugs: EO9; mitomycin C: bladder cancer; NQO1

Bladder cancer accounts for approximately 2% of all malignant cancers and is the fifth and tenth most common cancer in men and women respectively. The American Cancer Society estimated that 54 500 new cases and 11 700 deaths would have occurred in 1997. Superficial bladder cancer (pTa, pT1 and CIS) accounts for 70–80% of cancers at first presentation. Management of superficial bladder cancer is typically by endoscopic surgical resection often followed by a course of adjuvant intravesical chemotherapy or immunotherapy with the aim of both eradicating remaining tumour cells and preventing tumour recurrence (Herr, 1987). Both antineoplastics (Mitomycin C [MMC], epirubicin and thioTEPA) and immunotherapy (BCG) administered intravesically are effective at reducing tumour recurrence rates although it is unclear whether disease progression to muscle invasive tumours is prevented (Newling, 1990; Oosterlink et al, 1993). This observation in conjunction with the fact that mortality from bladder cancer is still high underscores the need to develop more effective therapeutic agents (Oosterlink et al, 1993).

MMC belongs to a class of compounds known as bioreductive drugs (Workman, 1994) and represents one of the antineoplastic agents used to treat superficial bladder cancers (Maffezzini et al, 1996; Tolley et al, 1996). MMC is activated to a cytotoxic species by cellular reductases although the role of specific reductase enzymes involved in bioreductive activation remains poorly defined and controversial (Cummings et al, 1998a). This is particularly true for the enzyme NQO1 (NAD(P)H:Quinone oxidoreductase, EC 1.6.99.2) which is a cytosolic flavoprotein which catalyses the two electron reduction of various quinone based compounds using either NADH or NADPH as electron donors (Schlager and Powis, 1988; Siegel et al, 1990). The structurally related compound EO9 (5-aziridinyl-3-hydroxymethyl-l-methyl-2-[1*H*-indole-4,7-dione]prop-β-en-α-ol), is however a much better substrate for NQO1 than MMC (Walton et al, 1991) and a good correlation exists between NQO1 activity and chemosensitivity in vitro under aerobic conditions (Robertson et al, 1994; Smitkamp-Wilms et al, 1994; Fitzsimmons et al, 1996). Under hypoxic conditions however, EO9's properties are markedly different with little or no potentiation of EO9 toxicity observed in NQO1 rich cells (Plumb and Workman, 1994). In NQO1-deficient cell lines however, large hypoxic cytotoxicity ratios have been reported (Workman, 1994). EO9 therefore has the potential to exploit the aerobic fraction of NQO1-rich tumours or the hypoxic fraction of NQO1-deficient tumours (Workman, 1994).

EO9 has been clinically evaluated but despite reports of three partial remissions in phase I clinical trials, no activity was seen against NSCLC, gastric, breast, pancreatic and colon cancers in subsequent phase II trials (Schellens et al, 1994; Dirix et al, 1996). These findings are particularly disappointing in view of the preclinical studies (Hendriks et al, 1993) together with reports that several tumour types have elevated NQO1 levels (Malkinson et al,

*Received 19 January 2001*
*Revised 3 June 2001*
*Accepted 4 July 2001*

*Correspondence to:* RM Phillips

1992; Smitkamp-Wilms et al, 1995; Siegel et al, 1998). Several possible explanations have been proposed to explain EO9's lack of clinical efficacy (Connors, 1996; Phillips et al, 1998). Recent studies have demonstrated that the failure of EO9 in the clinic may not be due to poor pharmacodynamic interactions but may be the result of poor drug delivery to tumours (Phillips et al, 1998). The rapid plasma elimination of EO9 ($t1/2 < 10$ min in humans) in conjunction with poor penetration through multicell layers suggests that EO9 will not penetrate more than a few microns from a blood vessel within its pharmacokinetic lifespan (Schellens et al, 1994; Phillips et al, 1998). Intratumoural administration of EO9 to NQO1-rich and -deficient tumours produced significant growth delays (although a distinction between damage to the aerobic or hypoxic fraction was not determined) suggesting that if EO9 can be delivered to tumours, therapeutic effects may be achieved (Cummings et al, 1998b). Whilst these undesirable characteristics are a serious setback for the treatment of systemic disease, paradoxically they may be advantageous for treating cancers which arise in a third compartment such as superficial bladder cancer. In this scenario, drug delivery is not problematical via the intravesical route and the penetration of EO9 into avascular tissue can be increased by maintenance of therapeutically relevant drug concentrations within the bladder (using a one hour instillation period for example). NQO1 activity in tumour tissue will be the principal determinant of selectivity of EO9 whether it is targeting the aerobic fraction (where high levels of NQO1 are desirable) or the hypoxic fraction of tumours (where low NQO1 and the presence of hypoxia are essential). The principal aim of this study therefore was to determine the activity of NQO1 in a series of human bladder tumours and normal bladder tissue by both enzymatic and immunohistochemical techniques. A secondary aim of this study was to evaluate a possible strategy for reducing possible systemic toxicity arising from intravesical therapy based upon the fact that the aerobic activity of EO9 against cell lines is enhanced under mild acidic conditions (Phillips et al, 1992). Administration of EO9 in an acidic vehicle would result in greater activity within the bladder and any drug absorbed into the blood stream would become relatively inactive due to the rise in extracellular pH. Selectivity for bladder tumours would still depend on tumour enzymology and therefore the second objective of this study was to determine the role of NQO1 in the activation of EO9 under acidic conditions.

### MATERIALS AND METHODS

### Collection of tumour and normal bladder specimens

Ethical approval for tissue collection was obtained from the Local Research Ethical Committee (Bradford NHS Trust) and samples taken from patients following informed consent. A total of 17 paired cold pinch biopsies were taken from bladder tumours and macroscopically normal-looking bladder mucosa at cystoscopy, immediately prior to formal transurethral resection of the tumour. Three specimens were taken from patients undergoing cystectomy and tumour and normal samples dissected by pathologists within 1 hour of surgical removal. Specimens were flash frozen in liquid nitrogen and transported for NQO1 enzyme analysis. Further biopsies were taken of the normal bladder mucosa immediately adjacent to the previous biopsy site and sent at the end of the procedure, along with the resected tumour, in formalin for routine histological analysis. In this way bladder tumour and normal bladder urothelium enzymology could be directly correlated with the appropriate tissue histology in each patient. Immunohistochemistry was performed from the subsequently archived wax blocks prepared for histology.

### Biochemical determination of NQO1 activity

Cell cultures in exponential growth were trypsinised, washed twice with Hanks balanced salt solution (HBSS) and sonicated on ice ($3 \times 30$ s bursts at 40% duty cycle and output setting 4 on a Semat 250 cell sonicator). NQO1 activity and protein concentration was determined as described below. Tissues were homogenised (10% w/v homogenate) in sucrose (0.25 M) using a 1 ml tissue homogeniser (Fisher Scientific). Cytosolic fractions were prepared by centrifugation of the homogenate at 18 000 ***g*** for 4 min followed by further centrifugation of the supernatant at 110 000 ***g*** for 1 h at 4˚C in a Beckman Optima™ TL ultracentrifuge. Activity of NQO1 in the supernatant was determined spectrophotometrically (Beckman DU650 spectrophotometer) by measuring the dicumarol sensitive reduction of dichlorophenolindophenol (DCPIP, Sigma Aldrich, UK) at 600 nm (Traver et al, 1992). This assay has been extensively validated for use in measuring NQO1 activity in both tissue and cell homogenates and has been shown to be preferable to other assays for NQO1 activity (Hodnick and Sartorelli, 1997). Each reaction contained NADH (200 µM), DCPIP (40 µM, Sigma Aldrich, UK), Dicumarol (20 µM, when required, Sigma Aldrich, UK), cytosolic fraction of tissues (50 µl per assay) in a final volume of 1 ml Tris HCl buffer (50 mM, pH 7.4) containing bovine serum albumin (0.7 mg $ml^{-1}$, Sigma Aldrich, UK). Rates of DCPIP reduction were calculated from the initial linear part of the reaction curve (30 s) and results were expressed in terms of nmol DCPIP reduced $min^{-1}$ $mg^{-1}$ protein using a molar extinction coefficient of 21 $mM^{-1}$ $cm^{-1}$ for DCPIP. Protein concentration was determined using the Bradford assay (Bradford, 1976).

### Immunohistochemistry

Polyclonal antibodies (raised in rabbits) to purified rat NQO1 were a gift from Professor Richard Knox (Enact Pharma Plc). Validation of the antibody for use in immunohistochemistry studies was performed by Western blot analysis using both purified human recombinant NQO1 and cell extracts derived from a panel of cell lines of human origin. These cell lines included H460 (human NSCLC), RT112 (human bladder carcinoma). HT-29 (human colon carcinoma). BE (human colon carcinoma). MT1 (human breast) and DLD-1 (human colon carcinoma). The BE cell line has been genotyped for the C609T polymorphic variant of NQO1 and is a homozygous mutant (and therefore devoid of NQO1 enzyme activity) with respect to this polymorphism (Traver et al, 1992). Cells were washed in ice-cold phosphate-buffered saline and lysed by sonication (30 s on ice) in Tris HCl (50 mM, pH 7.5) containing 2 mM EGTA, 2 mM PMSF and 25 µg $ml^{-1}$ leupeptin. Protein concentration was estimated using the Bradford assay (Bradford, 1976) and a total of 12.5 µg of protein (in Lamelli sample loading buffer) applied to a 12% SDS-PAGE gel. Following electrophoretic transfer to nitrocellulose paper, membranes were blocked in TBS/Tween 20 (0.1%) containing 5% non-fat dry milk for 1 h at room temperature. Membranes were washed in TBS/Tween 20 (0.1%) prior to the addition of rabbit anti-rat NQO1 antibody (1:100 dilution) and incubated at room temperature for 1 h.

 *© 2001 Cancer Research Campaign*

Membranes were extensively washed in TBS/Tween 20 (0.1%) followed by the addition of anti-rabbit IgG horseradish peroxidase conjugated secondary antibody (1:5000 dilution in TBS/Tween 20). Proteins were visualised by ECL-based chemiluminescence as described by the manufacturer (Amersham Pharmacia Biotech, Bucks, UK).

For immunohistochemical studies, all tissues (both tumour and normal bladder mucosa) were fixed in 10% formalin, processed routinely and embedded in paraffin wax. Two sections of each tissue block were placed on one slide, one section served as the test and the other as a negative control (no primary antibody). A total of 5 sections from each sample were stained for NQO1 (plus negative controls) and tumour and normal samples from a total of 17 patients were analysed. Sections (5 μm) were dewaxed, rehydrated and incubated with primary antibody (1:400 dilution) for 4 hours. Sections were then washed and incubated with biotinylated mouse anti-rabbit IgG for 30 min prior to immunoperoxidase staining using VECTASTAIN ABC reagents and DAB (Vector Laboratories Ltd, Peterborough, UK). Sections were counterstained with haematoxylin according to standard procedures.

### Cell culture and chemosensitivity studies

EO9 was a gift from NDDO Oncology, Amsterdam and MMC was obtained from the Department of Pharmacy, St Lukes Hospital, Bradford. H460 (human NSCLC) cell line was obtained from the American Type Culture Collection (ATCC). HT-29 (human colon carcinoma), RT112/83 (human bladder carcinoma epithelial), EJ138 (human bladder carcinoma) and T24/83 (human bladder transitional cell carcinoma) cell lines were obtained from the European Collection of Animal Cell Cultures (ECACC). A2780 (human ovarian carcinoma) and BE (human colon carcinoma) cells were gifts from Dr T Ward (Paterson Institute, Manchester, UK). All cell lines were maintained as monolayer cultures in RPMI 1640 culture medium supplemented with fetal calf serum (10%), sodium pyruvate (2 mM), L-glutamine (2 mM), penicillin/streptomycin (50 IU $ml^{-1}$ 50 μg $ml^{-1}$) and buffered with HEPES (25 mM). All cell culture materials were purchased from Gibco BRL (Paisley, UK). Cells were exposed to MMC or EO9 at a range of doses for 1 h and chemosensitivity was assessed following a 5 day recovery period using the MTT assay, details of which have been described elsewhere (Phillips et al, 1992). The pH of the medium used during drug exposure was adjusted using small aliquots of concentrated HCl (40 μl conc HCl (10.5 M) to 20 ml medium gives a pH of 6.0). Calibration curves were conducted over a broad range of pH values in culture medium (pH 3.5 to 11) and the stability of the pH conditions monitored over a 1 h incubation period at 37˚C. At all pH values, no significant changes in the pH of the medium was observed over the 1 h drug exposure period (data not presented).

HT-29 multicell spheroids were prepared by seeding $5 \times 10^5$ cells into T25 flasks which had been based coated with agar (1% w/v) and incubated for 24 h at 37˚C. Immature spheroids were then transferred to a spinner flask (Techne) containing 250 ml of RPMI 1640 growth medium and spheroids were kept in suspension by stirring at 50 rpm. When spheroids reached a diameter of approximately 500 μm, they were harvested for chemosensitivity studies. Multicell spheroids were exposed to a range of EO9 concentrations at pHe 6.0 and 7.4 for 1 h at 37˚C. Following drug incubation, spheroids were washed twice in HBSS prior to disaggregation into single cells using trypsin EDTA. Disaggregated spheroids were then washed in HBSS and then plated into 96 well plates ($1 \times 10^3$ cells per well) and incubated at 37˚C for 4 days. Chemosensitivity was assessed using the MTT assay as described elsewhere (Phillips et al, 1992).

The role of NQO1 in the activation of EO9 at pHe values of 7.4 and 6.0 was evaluated using the NQO1 inhibitor Flavone Acetic Acid (FAA), details of which are described elsewhere (Phillips, 1999). FAA is a competitive inhibitor of NQO1 with respect to NADH and at a final concentration of 2 mM, inhibition of NQO1 is >95% whereas the activity of cytochrome P450 reductase and cytochrome b5 reductase is not substantially altered (< 5% inhibition). Briefly, H460 cells (NQO1 rich) were plated into 96 well plates at a density of $2 \times 10^3$ cells per well. Following an overnight incubation at 37˚C, medium was replaced with fresh medium (pH 7.4) containing a non-toxic concentration of FAA (2 mM) and incubated for 1 h at 37˚C. Medium was then replaced with fresh medium containing EO9 (range of drug concentrations) and FAA (2 mM) at either pHe 7.4 or 6.0. Following a further 1 h incubation at 37˚C, cells were washed twice with HBSS and incubated at 37˚C in growth medium for 5 days. Chemosensitivity was determined by the MTT assay as described above and results were expressed in terms of $IC_{50}$ values, selectivity ratios ($IC_{50}$ at pHe 7.4/$IC_{50}$ at pHe 6.0) and protection ratios ($IC_{50}$ FAA/EO9 combinations/$IC_{50}$ for EO9 alone).

### Substrate specificity

The influence of acidic pHe on substrate specificity for purified human NQO1 was determined as described previously (Walton et al, 1991; Phillips 1996). NQO1-mediated reduction of the quinone to the hydroquinone species is difficult to detect by conventional assays thereby necessitating the use of a reporter signal generating step. In this assay, the hydroquinone acts as an intermediate electron acceptor which subsequently reduces cytochrome c which can readily be detected spectrophotometrically. Recombinant human NQO1 was derived from *E. coli* transformed with the pKK233-2 expression plasmid containing the full length cDNA sequence for human NQO1 (Beall et al, 1994). Following IPTG induction, NQO1 was purified by cybacron blue affinity chromatography, details of which are described elsewhere (Phillips, 1996). The purified protein had a molecular weight of approximately 31 kDa and a specific activity of 139 μmol DCPIP reduced $min^{-1}$ $mg^{-1}$ protein (Phillips, 1996). Reduction of EO9 by recombinant human NQO1 was determined at pH 6.0 and 7.4 by measuring the rate of reduction of cytochrome c at 550 nm on a Beckman DU 650 spectrophotometer according to previously published methods (Phillips, 1996). Results were expressed in terms of μmol cytochrome c reduced $min^{-1}$ $mg^{-1}$ protein using a molar extinction coefficient of 21.1 $mM^{-1}$ $cm^{-1}$ for cytochrome c.

### Measurement of intracellular pH

Intracellular pH was determined using the fluorescent pH indicator BCECF (2,7-bis-(2-carboxy-ethyl)-5-(and-6) ) carboxyfluorescein (Molecular Probes, Eugene, USA) according to the manufacturer's instructions. Confluent flasks of cells were washed with HBSS to remove any traces of serum containing RPMI medium and then incubated with the esterified form of BCECF (BCECF-AM) at a concentration of 2 μM in HBSS for 1 h at 37˚C. The non-denaturing detergent Pluronic was added to the probe to aid dispersion. Cells were then washed to remove all traces of BCECF-AM and then trypsinized before being suspended in serum-free/phenol red-free RPMI medium (Gibco BRL, Paisley, UK) at a concentration of $10^6$ cells $ml^{-1}$ at pH 6 for 1 h. Flourescence measurement was determined in a Perkin–Elmer fluorescence spectrophotometer in UV grade disposable 4 ml cuvettes (Fischer Scientific) with excitation wavelengths 500 nm and 450 nm (excitation bandpass slit of 10 nm) and emission wavelength fixed at 530 nm (emission bandpass slit of 2.5 nm). These were determined to be optimal settings for the machine and system under study. An in-situ calibration was performed for every pHi determination with a range of 6 pHs from 4 to 9 using the ionophore nigericin at a concentration of 22.8 µM to equilibrate pHe with pHi. Calculation of the ratio of fluorescence at 500 nm/450 nm was calculated after subtraction of background fluorescence from blanks at each pH (serum free, phenol red free RPMI without cells).

### RESULTS

### Activity of NQO1 in tumour and normal bladder specimens

The biochemical activity of NQO1 in paired samples of tumour (grade/stage ranging from G2 pTa to G2/G3 T4) and normal bladder mucosa (with 3 cystectomy specimens) taken from a series of 20 patients is presented in Table 1. Within the tumour specimens, a broad range of NQO1 activity existed ranging from 571.4 nmol $min^{-1}$ $mg^{-1}$ to undetectable (<0.1 nmol $min^{-1}$ $mg^{-1}$). In histologically normal bladder mucosa specimens, NQO1 activity ranged from 190.9 to < 0.1 nmol $min^{-1}$ $mg^{-1}$. In the majority of patients NQO1 activity in the tumour was greater than in the normal bladder mucosa. Tumour grade and stage did not correlate with NQO1 activity (Table 1).

### Validation of NQO1 antibody and immunohistochemical localisation of NQO1

Western blot analysis demonstrates that polyclonal anti-rat NQO1 antibody cross reacts with human NQO1 (Figure 1) with a single band at approximately 31 kDa observed for both cell extracts and purified human NQO1. Titration of purified NQO1 results in a decrease in band intensity (Figure 1B) and in cell extracts, band intensity was qualitatively consistent with NQO1 enzyme activity (Figure 1A). In addition, the antibody does not detect NQO1 in the BE cell line which is devoid of NQO1 activity as a result of the C609T polymorphism (Figure 1C). No non-specific bands were observed on Western blots. Immunoperoxidase staining of NQO1 protein in tumour tissue, bladder wall, ureter and urethra are presented in Figure 2. Superficial and invasive tumours (pTa – (**A**); G3 pT2 – (**B**); G3pT4 – (**C**) ) with high to intermediate levels of NQO1 as determined by biochemical assays (patient numbers 1, 4 and 5 in Table 1) clearly stained positive for NQO1. Staining was confined to the cytoplasm of tumour cells with little or no staining of stromal cells (panels B and C). In other tumours with intermediate or low levels of NQO1 activity, staining was heterogeneous with pockets of cells containing high levels of NQO1 protein (data not shown). Normal bladder wall sections were obtained from a patient who underwent cystectomy (G3pT4 bladder tumour), ureter and urethra were obtained from another patient who underwent cystectomy (G3 pT3a bladder tumour). In the bladder wall, no NQO1 staining was observed in the urothelium (**D**) although slight staining was present in smooth muscle layers. The urethra (**E**) was negative although cells on the luminal surface of the ureter were positively stained (**F**). The basal layers of the ureter lining were however negatively stained (**F**). No evidence of invasive malignancy or in-situ carcinoma were observed in the ureter and urethra or in the section of bladder wall presented (**D**). In 16 other normal bladder biopsy and cystectomy specimens, no positive staining of the urothelium was observed (data not shown).

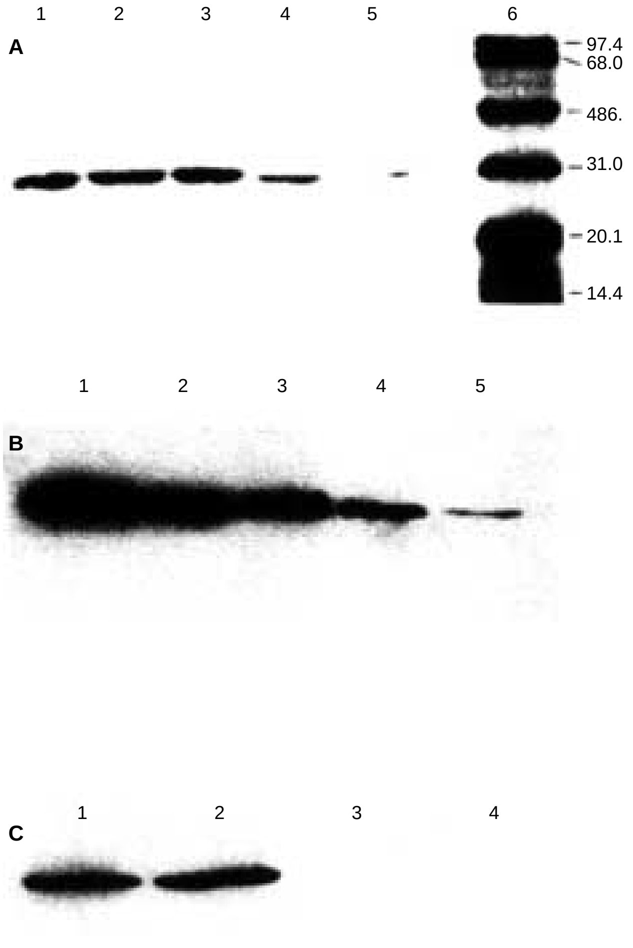

**Figure 1** Validation of the polyclonal anti-rat NQO1 antibody for use in immunohistochemical analysis of human NQO1. (**A**) Western blot analysis of cell extracts (12.5 µg protein loaded per lane) for NQO1. Lanes 1–5 represent extracts from DLD-1 (794 ± 121 nmol $min^{-1}$ $mg^{-1}$), HT-29 (688 ± 52 nmol $min^{-1}$ $mg^{-1}$), H460 (1652 ± 142 nmol $min^{-1}$ $mg^{-1}$), MT1 (287 ± 53 nmol $min^{-1}$ $mg^{-1}$), and RT112 (30 ± 3 nmol $min^{-1}$ $mg^{-1}$) respectively where the values in parenthesis represent NQO1 activity. Lane 6 represents molecular weight markers (ECL protein molecular weight markers, Amersham Pharmacia Biotech, UK). (**B**) Western blot analysis using purified human recombinant NQO1. Lanes 1–5 represent protein amounts of 0.25, 0.125, 0.0625, 0.0312 and 0.0156 pmol respectively. (**C**) Western blot analysis of cell extracts (25 µg protein loaded per lane) derived from H460 cells (lanes 1–2) and BE cells (lanes 3–4)

### Influence of pH on substrate specificity and chemosensitivity

The ability of EO9 to serve as a substrate for NQO1 was not influenced by pH with specific activities of 21.10 ± 2.3 and 21.30 ± 1.5 µmol cytochrome c reduced $min^{-1}$ $mg^{-1}$ protein at pH 7.4 and 6.0 respectively. The response of a panel of cell lines with a range of

 *© 2001 Cancer Research Campaign*

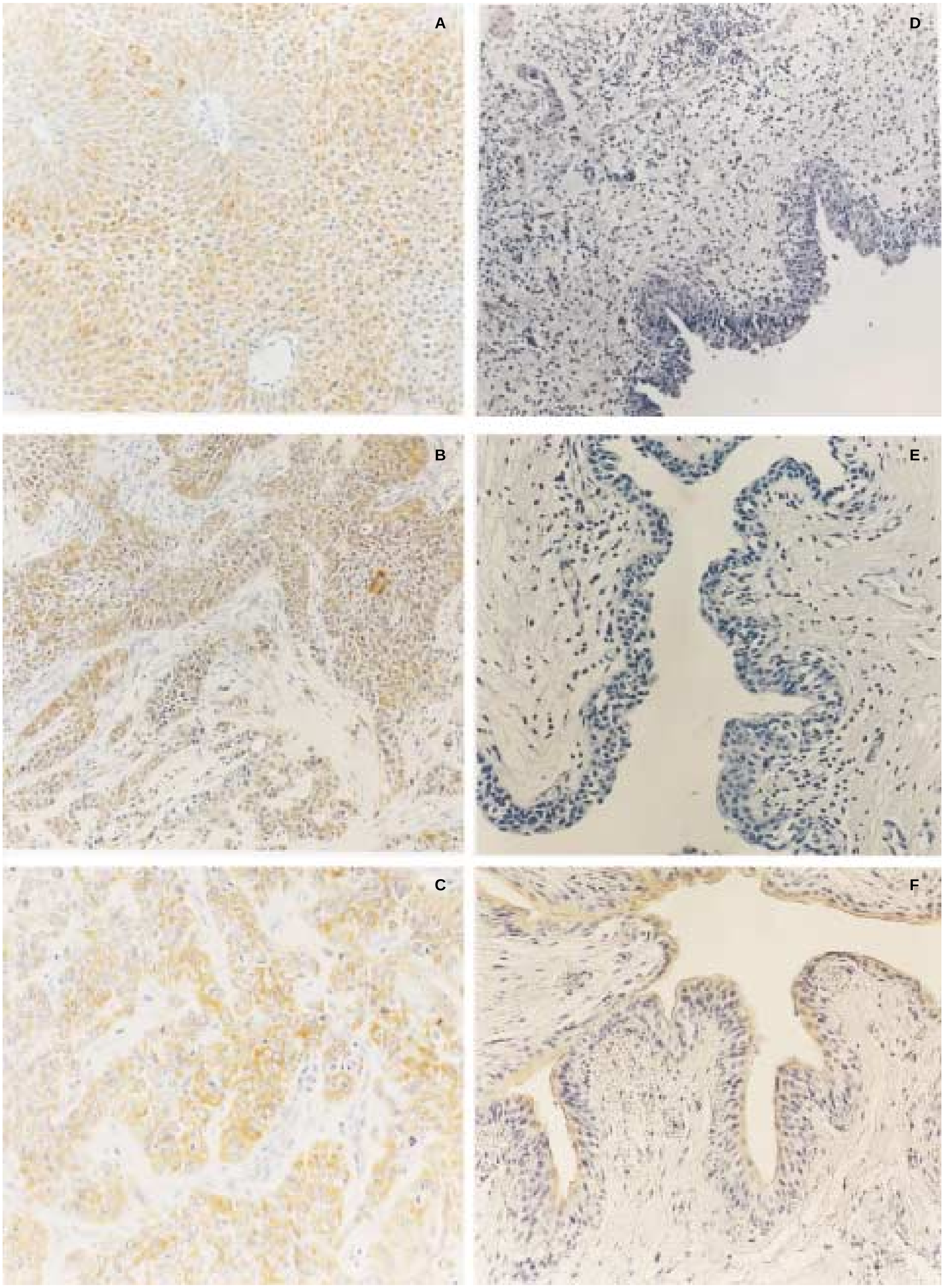

**Figure 2** Immunohistochemical localisation of NQO1 in human bladder tumours, normal bladder, urethra and ureter. Tumours (**A**, **B** and **C**) were classified as G2 pTa (**A**, [× 200]) and G3 pT2 (**B** [× 100]) and G3 pT4 (**C** [× 200]) which had high to intermediate levels of NQO1 activity as determined by biochemical methods. (**D**) (× 100) represents a histological section through a macroscopically normal-looking section of bladder from a patient who underwent cystectomy for a G3 pT4 tumour; no tumour was identified in these sections but some inflammatory change was evident. (**E**) and (**F**) (× 200) represent urethra and ureter with no evidence of invasive or in situ carcinoma in these sections. All sections have been stained with NQO1 antibody. Negative staining (without primary antibody) were clear (data not shown)

**Table 1** Tumour histology reports and NQO1 activity in paired samples of bladder tumour and normal bladder mucosa

| Patient no. | Tumour histology | NQO1 Activity Tumour (nmol min$^{-1}$ mg$^{-1}$) | NQO1 Activity Normal (nmol min$^{-1}$ mg$^{-1}$) | Ratio of NQO1 levels in tumour to normal tissue |
|---|---|---|---|---|
| 1[f,s,i,p] | G2 pTa | 571.4 | < 0.1 | 571.40 |
| 2[m,s,r] | G3 pT2 | 273.3 | < 0.1 | 273.30 |
| 3[f,s,i] | G1 pTa | 107.80 | < 0.1 | 107.80 |
| 4[m,e,i] | G3 pT2/3 | 73.36 | < 0.1 | 73.36 |
| 5[m,s,i] | G3 pT4 (C) | 81.30 | 4.10 | 19.83 |
| 6[h] | G2 pT1 | 309.50 | 25.20 | 12.10 |
| 7[m,n,r,o] | G3 pT2 | 10.00 | < 0.1 | 10.00 |
| 8[f,n,i] | G3 pT2 | 9.80 | < 0.1 | 9.80 |
| 9[m,n,i] | G2 pT2 | 4.40 | < 0.1 | 4.40 |
| 10[m,s,c] | G3 pT2 | 34.01 | 8.50 | 4.00 |
| 11[m,s] | G1 pTa | 69.76 | 22.20 | 3.14 |
| 12[m,n] | G1 pTa | 42.16 | 15.30 | 2.73 |
| 13[m,n,i] | G3 pT2 | 179.6 | 72.12 | 2.49 |
| 14[m,e,i] | G2/G3 T4 (C) | 89.70 | 63.30 | 1.41 |
| 15[m,n,r] | G3 pT2 | 0.40 | < 0.1 | 0.40 |
| 16[m,e,c,o] | G3 pT3 (C) | 21.60 | 61.70 | 0.35 |
| 17[f,n,i] | G2 pT1 | 58.40 | 190.90 | 0.30 |
| 18[m,e,o] | G2 pT1 | < 0.1 | < 0.1 | 0 |
| 19[f,n,i] | G2 pT1 | < 0.1 | < 0.1 | 0 |
| 20[m,e,c,r] | G2 pT0 | < 0.1 | < 0.1 | 0 |

[m]Male, [f]Female, [s]Smoker, [n]Non-smoker, [e]Ex-smoker, [c]Intravesical chemotherapy prior to specimen collection, [r]Radiotherapy prior to specimen collection, [i]First presentation, [p]Previous malignancy other than bladder, [h]No medical history available, [o]Possible occupational carcinogen exposure (ie dye industry worker). (C) denotes cystectomy specimens. In all cases, protein levels following preparation of the cytosolic fraction were greater than 0.1 mg ml$^{-1}$.

**Table 2** The relationship between NQO1 activity and chemosensitivity to EO9 and MMC under physiological and acidic pHe conditions

| Cell line | Drug | NQO1 (nmol min$^{-1}$ mg$^{-1}$) | $IC_{50}$pHe 7.4 (nM) | $IC_{50}$ pHe 6.0 (nM) | SR* |
|---|---|---|---|---|---|
| H460 | EO9 | 1652 ± 142 | 60 ± 10 | 9.5 ± 2 | 6.31 |
| HT-29 | EO9 | 688 ± 52 | 120 ± 53 | 29 ± 10 | 4.13 |
| T24/83 | EO9 | 285 ± 28 | 290 ± 65 | 60 ± 18 | 4.83 |
| A2780 | EO9 | 159 ± 33 | 200 ± 50 | 51 ± 14 | 3.92 |
| EJ138 | EO9 | 83 ± 14 | 310 ± 95 | 39 ± 7 | 7.94 |
| RT112 | EO9 | 30 ± 3 | 1050 ± 75 | 61 ± 13 | 17.21 |
| BE | EO9 | < 0.1 | 5300 ± 169 | 1300 ± 75 | 4.07 |
| H460 | MMC | 1652 ± 142 | 900 ± 200 | 220 ± 130 | 4.50 |
| HT-29 | MMC | 688 ± 52 | 1050 ± 210 | 500 ± 240 | 2.10 |
| T24/83 | MMC | 285 ± 28 | 2150 ± 93 | 2100 ± 800 | 1.02 |
| A2780 | MMC | 159 ± 33 | 2400 ± 340 | 1400 ± 130 | 1.71 |
| EJ138 | MMC | 83 ± 14 | 1600 ± 200 | 1400 ± 250 | 1.14 |
| RT112 | MMC | 30 ± 3 | 3350 ± 250 | 2000 ± 500 | 1.67 |
| BE | MMC | < 0.1 | 7000 ± 192 | 4400 ± 215 | 1.59 |

All results presented are the mean of 3 independent experiments (SD values omitted in the interests of presentation). *SR (selectivity ratio) = $IC_{50}$ at pH 7.4/$IC_{50}$ at pH 6.0.

NQO1 activity (<1.0 to 1898 ± 276 nmol min$^{-1}$ mg$^{-1}$) to EO9 and MMC at pHe values of 7.4 and 6.0 is presented in Table 2 and Figure 2. At pHe = 7.4, a good correlation existed between NQO1 activity and chemosensitivity to EO9 (Figure 3). In the case of MMC (Table 2, Figure 3), a relationship between NQO1 and chemosensitivity was apparent (at pHe 7.4) although this relationship was not as prominent as shown by EO9 with a narrow range of $IC_{50}$ values (range 0.9 to 7.0 μM) observed in cell lines which cover a broad range of NQO1 activity (ranging from < 1.0 to 1652 nmol min$^{-1}$ mg$^{-1}$). Both MMC and EO9 are preferentially more toxic to cells at pHe values of 6.0 although much greater potentiation of EO9 activity is seen with SR values (SR = selectivity ratio defined as $IC_{50}$ pHe 7.4/$IC_{50}$ pHe 6.0) ranging from 3.92 to 17.21 for EO9 compared with 1.02 to 4.50 for MMC (Table 2). The activity of EO9 was enhanced in both NQO1 rich and deficient cell lines when pHe was reduced to 6.0 and the relationship between NQO1 and chemosensitivity remained good when cells were exposed to EO9 under acidic conditions (Figure 3). No cell

 *© 2001 Cancer Research Campaign*

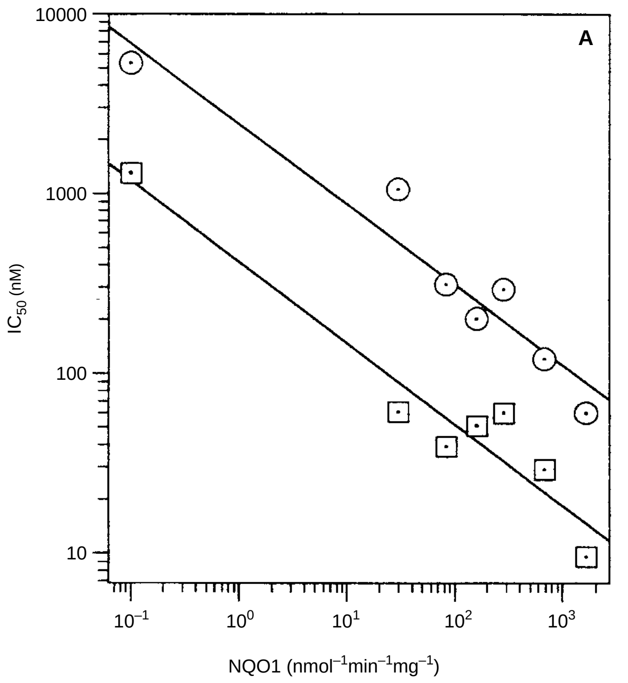

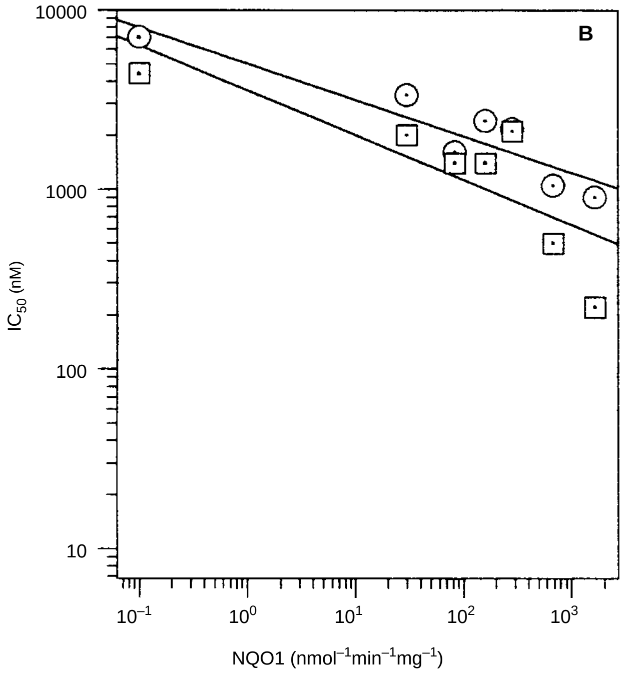

**Figure 3** The relationship between NQO1 activity and the response of a panel of cell lines to EO9 (**A**) or MMC (**B**) under normal physiological pHe of 7.4 (○) or acidic pHe values of 6.0 (□). Regression analysis data (as determined by Sigma Plot graphics) for EO9 at pH 7.4 were r = 0.886, slope = –0.52 and at pH 6.0, regression analysis data for EO9 was r = 0.804 and slope = –0.51. For MMC, regression analysis at pH 7.4 was r = 0.849, slope = –0.19 and at pH 6.0, r = 0.609, slope = –0.23

kill was observed in control cultures when the pHe was decreased to 6.0 (in the absence of drug) as determined by the MTT assay. The response of H460 cells to EO9 at pHe values of 7.4 and 6.0 in the presence and absence of FAA (2 mM) is presented in Table 3. At both pHe values, the response of H460 cells to EO9 was reduced in the presence of FAA. Protection ratios defined as the $IC_{50}$ for EO9 plus FAA divided by the $IC_{50}$ value for EO9 alone were similar for cells under acidic and physiological pHe values (14.63 and 13.95 respectively, Table 3). Selectivity ratios defined as the $IC_{50}$ at pHe 7.4 divided by the $IC_{50}$ at pHe 6.0 in the presence and absence of FAA were also similar with SR values of 6.31 and 6.02 for EO9 alone and EO9 plus FAA respectively (Table 3). The response of HT-29 multicell spheroids to EO9 is presented in Figure 4. Spheroids exposed to EO9 at pHe 6.0 were significantly more responsive than at pHe 7.4 with $IC_{50}$ values of 9.89 ± 0.89 and 24.24 ± 3.29 µM respectively. Spheroids were significantly

**Table 3** Response of H460 cells to EO9 in the presence or absence of FAA (2 mM) at pHe values of 7.4 and 6.0

| Drug | pHe | $IC_{50}$ (nM) | SR* | PR** |
|---|---|---|---|---|
| EO9 | 7.4 | 60.0 ± 8.1 | – | – |
| EO9 | 6.0 | 9.5 ± 2.6 | 6.31 | – |
| EO9/FAA | 7.4 | 837 ± 45 | – | 13.95 |
| EO9/FAA | 6.0 | 139 ± 27 | 6.02 | 14.63 |

*SR = Selectivity ratio defined as the ratio of $IC_{50}$ values at pHe = 7.4 divided by the $IC_{50}$ at pHe = 6.0. **PR = Protection ratio defined as the ratio of $IC_{50}$ values for EO9 plus FAA divided by the $IC_{50}$ values for EO9 alone. All values represent the mean ± standard deviation for three independent experiments.

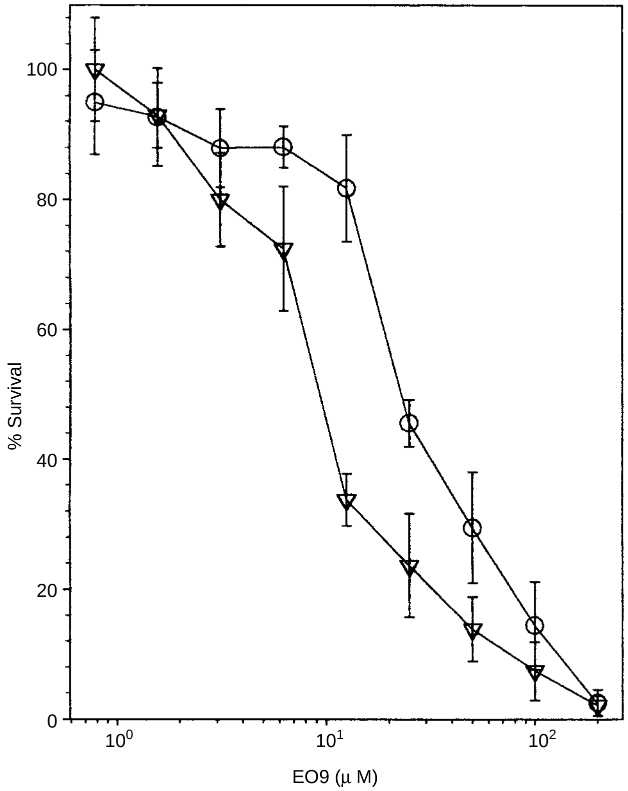

**Figure 4** Response of HT-29 multicell spheroids following a one hour exposure to EO9 under acidic (pHe = 6.0, ∇) and physiological (pHe = 7.4, O) extracellular pH conditions. Values presented are the means of 3 independent experiments ± standard deviation less responsive to EO9 than the same cells exposed to EO9 as monolayers at both pHe values with ratios of $IC_{50}$ values for spheroids to monolayers of 202 and 341 at pHe values of 7.4 and 6.0, respectively.

### Influence of acidic pHe conditions on pHi

PHi values following a 1 h incubation at pHe 6.0 were 6.44 ± 0.04, 6.51 ± 0.02 and 6.42 ± 0.05 in A549, RT112/83 and A2780 cells, respectively. Addition of the ionophore nigericin (after a 1 h incubation at pHe 6.0) resulted in the equilibration of pHe and pHi.

### DISCUSSION

In terms of bioreductive drug development, two of the critical factors which will ultimately determine selectivity are the enzymology of tumours and the presence of hypoxia (Workman, 1994). As outlined in the introduction, the presence or absence of NQO1 is central to the design of appropriate EO9-based therapeutic strategies aimed at targeting either the aerobic (NQO1-rich cells) or hypoxic (NQO1-deficient tumours) fraction of tumours. Workman (1994) has outlined a proposed mechanism for the different properties of EO9 under aerobic and hypoxic conditions based on the hypothesis that it is the semiquinone (product of one electron reduction) rather than the hydroquinone which is responsible for toxicity. In NQO1-deficient cells, the semiquinone produced as a result of one electron reductases would be relatively non-toxic as it would rapidly redox cycle back to the parent compound. Free radical species generated as a result of redox cycling would be detoxified by superoxide dismutase or catalase but under hypoxic conditions, the semiquinone would be relatively stable. If this were the major toxic species, then the activity of EO9 against cells with low NQO1 would be potentiated. In NQO1-rich cells however, the major product formed would be the hydroquinone. Aerobic toxicity could be generated as a result of the back oxidation of the hydroquinone to the semiquinone species or the parent quinone (Butler et al, 1996) resulting in free radical generation. Under hypoxic conditions however the hydroquinone will be more stable and if this is relatively non-toxic, then the activity of EO9 against NQO1 cells under hypoxia would not be potentiated. Whilst the mechanism of action of EO9 under aerobic and hypoxic conditions is complex, the biological data suggest that EO9 should target the aerobic fraction of NQO1-rich tumours or the hypoxic fraction of NQO1-deficient tumours (Workman, 1994).

Analysis of NQO1 activity in tumour and normal bladder tissues has clearly identified patients whose tumours are either NQO1-rich or NQO1-deficient (Table 1). Within the subset of NQO1-rich tumours, enzyme activity is elevated relative to the normal bladder urothelium. Immunohistochemical studies confirm these biochemical measurements with staining confined to tumour cells as opposed to normal stromal cells (Figure 2A, B and C). Within normal bladder tissues, NQO1 staining was absent from the urothelial lining of the bladder (Figure 2D) and the urethra (Figure 2E). Faint staining of the superficial layers of the ureter (Figure 2F) was observed although the underlying basal layers of the ureter were negatively stained. Similarly, faint staining of the smooth muscle layers of the bladder, ureter and urethra were also observed (data not shown). These studies suggest that a proportion of patients with bladder tumours (at various grades and stages of the disease) exhibit a significant differential in terms of NQO1 activity which could potentially be exploited by EO9-based therapies directed against the aerobic fraction of tumour cells. With regards to the ability of EO9 to selectively kill hypoxic NQO1-deficient cells, a subset of patients also exist whose tumours are devoid of NQO1 activity (Table 1). It is not known whether or not bladder tumours contain regions of low oxygen tension and further studies are required using hypoxia markers such as pimonidazole (Kennedy et al, 1997) to address this issue and to establish the relationship between NQO1 activity and hypoxia in tumours.

Whilst biochemical and immunohistochemical studies demonstrate that a subset of patients exist which have the appropriate tumour enzymology to activate EO9 (under aerobic conditions), intravesical chemotherapy can result in systemic toxicity due to the drug entering the blood supply. This study has also evaluated a potential strategy for minimising any risk of systemic toxicity based upon the hypothesis that administration of EO9 in an acidic vehicle would enhance the potency of EO9 (Phillips et al, 1992) within the bladder and that any drug reaching the blood stream would become relatively inactive due to a rise in pHe. Selectivity for aerobic cells would still be determined by NQO1 activity and therefore it is essential to determine the role that NQO1 plays in the activation of EO9 under acidic pHe conditions. In a panel of cell lines with a broad spectrum of NQO1 activity, reducing the pHe to 6.0 enhances the potency of EO9 under aerobic conditions in all cases (with SR values ranging from 3.92–17.21, Table 2). In the case of MMC, potency is also enhanced at low pHe values although the magnitude of the pH-dependent increase in toxicity is reduced (SR values ranging from 1.02–4.50, Table 2) compared with EO9. With respect to MMC, one explanation for increased activity under acidic conditions has been attributed to the fact that MMC becomes a substrate for NQO1 under acidic conditions (Pan et al, 1993; Siegel et al, 1993). This is not the case with EO9 as rates of reduction of EO9 by purified human NQO1 are not influenced by pH (21.10 ± 2.30 and 21.30 ± 1.50 μmol cytochrome c reduced $min^{-1}$ $mg^{-1}$ protein at pH 7.4 and 6.0, respectively). Recent studies have demonstrated that the activity of EO9 is enhanced under acidic conditions (pHe = 6.5) but only when the intracellular pH is reduced (pHi = 6.5) by co-incubation with nigericin (Kuin et al, 1999). The results of this study are in agreement with this finding as pHi becomes acidic (pHi values range from 6.42 ± 0.05 to 6.51 ± 0.02 depending on the cell line) when cells are cultured under pHe 6.0 conditions.

In the panel of cell lines used in this study, a good correlation exists between NQO1 activity and chemosensitivity at both pHe values of 7.4 and 6.0 (Figure 3). A strong relationship between NQO1 activity and response under aerobic conditions (at pHe 7.4) has been established previously by several groups (Robertson et al, 1994; Smitkamp-Wilms et al, 1994; Fitzsimmons et al, 1996) and there is clear evidence that NQO1 plays a central role in the mechanism of action of EO9 under aerobic conditions (Workman, 1994). The good correlation between NQO1 activity and response at pHe 6.0, in conjunction with the fact that EO9 is still a good substrate for NQO1 at pH 6.0, suggests that NQO1 plays a significant role in EO9's mechanism of action at acidic pHe values under aerobic conditions. It is of interest to note however that the activity of EO9 against BE cells (which are devoid of NQO1 activity as a result of the C609T polymorphism; Traver et al, 1992) is also enhanced under acidic pHe conditions (Table 2). This suggests that there is a NQO1-independent mechanism for the increased activity of EO9 under acidic conditions. This is confirmed by the use of the NQO1 inhibitor FAA where the 'protection ratios' (defined as the

 *© 2001 Cancer Research Campaign*

ratio of $IC_{50}$ values for EO9 plus FAA divided by the $IC_{50}$ values for EO9) are similar at both pHe 7.4 and 6.0 (13.95 and 14.63, respectively, Table 3). If NQO1 played a central role in the activation of EO9 at pHe 6.0, then the protection ratio at pHe 6.0 would be significantly greater than the protection ratio at pHe 7.4. The mechanism behind the NQO1-independent activation of EO9 is unclear although it is a well known fact that the reactivity of aziridine ring structures is enhanced by protonation resulting in ring opening to the aziridinium ion which is a potent alkylating species (Mossoba et al, 1985; Gutierrez, 1989). Alternatively, EO9 is a substrate for other one electron reductases (Maliepaard et al, 1995; Saunders et al, 2000) and further studies designed to evaluate whether EO9's metabolism by these enzymes is pH dependent needs to be determined. The potency of EO9 can be enhanced further by reducing pHe below 6.0 (Phillips et al, 1992) but these conditions are unlikely to provide significant clinical benefits as EO9 becomes progressively more unstable when pH is reduced to 5.5 (t1/2 = 37 min). From a pharmacological standpoint, administration of EO9 in a vehicle at pH 6.0 would appear desirable. Not only would this result in significant enhancement of EO9 activity but also the stability of EO9 would be sufficient (t1/2 = 2.5 h) to maintain drug exposure parameters at a therapeutic level.

With regards to the activity of EO9 against three-dimensional culture models in vitro, this study has demonstrated that reducing the pHe to 6.0 enhances the potency of EO9 against multicell spheroids although the magnitude of this effect is reduced compared with monolayer cultures (Figure 4). It is not known whether or not reduction in pHe results in greater cell kill throughout the spheroid or if it is confined to the surface of the spheroid exposed to medium. In comparison with MMC, previous studies using histocultures exposed to MMC demonstrated that no difference in toxicity exists between physiological and acidic pHe conditions (Yen et al, 1996). The pH-dependent increase in EO9 toxicity against spheroids suggests that manipulation of pHe may not only be of use in treating a multilayered solid bladder tumour but may offer an advantage over MMC. It should however be stated that multicell spheroids are significantly less responsive to EO9 than monolayers, presumably because of the poor penetration properties of EO9 through avascular tissue (Phillips et al, 1998). EO9 can nevertheless kill >90% of cells in spheroids (Figure 4) suggesting that at higher doses at least, the penetration of EO9 is sufficient to eradicate cells which reside some distance away from the surface of the spheroid.

In conclusion, the results of this study have demonstrated that within a population of patients with bladder tumours at various stages and grades of the disease, there exists a great heterogeneity regarding the expression of NQO1. The majority of patients have tumours possessing elevated levels of NQO1 while a small subset of patients appear to be devoid of NQO1 activity. The heterogeneous nature of NQO1 activity described here is consistent with several other studies in various tumour types (Malkinson et al, 1992; Smitkamp-Wilms et al, 1995; Siegel et al, 1998). These findings reinforce the view that 'enzyme profiling' of individual patients could be valuable prior to therapeutic intervention with bioreductive drugs (Workman, 1994). This is to our knowledge the first study to characterise NQO1 activity and cellular localisation in bladder tumours and provide strong evidence to support the evaluation of EO9 against superficial and locally invasive bladder tumours. This study has clearly demonstrated that under aerobic conditions, EO9 is much more potent under acid conditions (pH 6.0) than at physiological pH (pH 7.4). The mechanism for this increased EO9 potency appears to be NQO1 independent and whilst this will not improve (or reduce) selectivity, it may prove beneficial in terms of reducing the therapeutically effective dose of EO9. Dose reduction in conjunction with the fact that a reduction in the potency of EO9 due to the increased pHe in the blood stream suggests that systemic toxicity arising from the intravesical administration of EO9 would be low. In addition, this study shows that under physiological conditions the activity of EO9 is much lower in tissues with 'normal' expression of NQO1 compared to 'high' NQO1-expressing tissues (i.e. the tumours). The results of this study provide strong evidence in support of the proposal that intravesical administration of EO9 may have activity against bladder tumours.

### ACKNOWLEDGEMENTS

The authors wish to acknowledge BP Cronin, AG Breen, CM Jarret (University of Bradford) and G Meah (Histopathology, Bradford NHS Trust) for technical assistance and Prof R Knox (Enact Pharma Plc, Porton Down, UK) for supplying the polyclonal antibody to NQO1. This work was supported by the Cancer Research Campaign (RMP and JAD programme grant number SP 2523/0101), Kyowa Hakko UK Ltd (GAC) and NDDO Oncology, Amsterdam.

### REFERENCES

Bradford MM (1976) A rapid and sensitive method for the quantification of microgram quantities of protein utilising the principle of protein-dye binding. *Anal Biochem* **72**: 248–254

Butler J, Spanswick VJ and Cummings J (1996) The autooxidation of reduced forms of EO9. *Free Rad Res* **25**: 141–148

Connors TA (1996) Bioreductive agents, hypoxic cells and therapy. *Eur J Cancer* **32A**: 1833–1834

Cummings J, Spanswick VJ, Tomaz M and Smyth JF (1998a) Enzymology of MMC metabolic activation in tumour tissue. Implications for enzyme directed bioreductive drug development. *Biochem Pharmacol* **56**: 405–414

Cummings J, Spanswick VJ, Gardiner J, Ritchie A and Smyth JF (1998b) Pharmacological and biochemical determinants of the antitumour activity of the indoloquinone EO9. *Biochem Pharmacol* **55**: 253–260

Dirix LY, Tonnesen F, Cassidy J, Epelbaum R, Huinink WWT, Pavlidis N, Sorio R, Gamucci T, Wolff I, Tevelde A, Lan J and Verweij J (1996) EO9 phase II study in advanced breast, gastric, pancreatic and colorectal carcinoma by the early clinical studies group. *Eur J Cancer* **32A**: 2019–2022

Fitzsimmons SA, Workman P, Grever M, Paull K, Camalier R and Lewis AD (1996) Reductase enzyme expression across the National Cancer Institute tumour cell line panel: Correlation with sensitivity to MMC and EO9. *J Natl Cancer Inst* **88**: 259–269

Gutierrez PL (1989) Mechanism of bioreductive alkylation. The example of diazaquinone (AZQ). *Free Radical Bio Med* **6**: 405–445

Hendriks HR, Piazo PE, Berger DP, Kooistra KL, Bibby MC, Boven E, Dreef-Van Der Meulen HC, Henrar REC, Fiebig HH, Double JA, Hornstra HW, Pinedo HM, Workman P and Swartsmann G (1993) EO9: A novel bioreductive alkylating indolequinone with preferential solid tumour activity and lack of bone marrow toxicity in preclinical models. *Eur J Cancer* **29A**: 897–906

Herr HW (1987) Intravesical therapy – a critical review. *Urol Clin N Am* **14**: 399–404

Hodnick WF and Sartorelli AC (1997) Measurement of dicumarol sensitive NADPH: (menadione cytochrome c) oxidoreductase activity results in an artifactual assay of DT-diaphorase in cell sonicates. *Anal Biochem* **252**: 165–168

Kennedy AS, Raleigh JA, Perez GM, Calkins DP, Thrall DE, Novotny DB and Varia MA (1997) Proliferation and hypoxia in human squamous cell carcinoma of the cervix: first report of combined immunohistochemical assays. *Int J Radial Oncol Biol Phys* **37**: 897–905

Kuin A, Alders M, Lamfers M, Van Zuidam DJ, Essers M, Beijnen JH and Smets LA (1999) Potentiation of anti-cancer activity at low intratumoural pH induced by the mitochondrial inhibitor *m*-iodobenzylguanidine (MIBG) and its analogue benzylguanidine (BG). *Br J Cancer* **79**: 793–801

*© 2001 Cancer Research Campaign* 

Maffezzini M, Simonata A, Zanon M, Raber M and Carmignani G (1996) Up-front chemotherapy for low stage low grade recurrent bladder cancer. *J Urol* **155**: 91–93

Malkinson AM, Siegel D, Forrest GL, Gazdar AF, Oie HK, Chan DC, Bunn PA, Mabry M, Dykes DJ, Harrison SD and Ross D (1992) Elevated DT-diaphorase activity and messenger RNA content in human non small cell lung carcinoma – Relationship to the response of lung tumour xenografts to MMC. *Cancer Res* **52**: 4752–4757

Maliepaard M, Wolf A, Groot SE, De Mol NJ and Janssen LHM (1995) Indoloquinone EO9: DNA interstrand cross linking upon reduction by DT-diaphorase or xanthine oxidase. *Br J Cancer* **71**: 836–839

Mossoba MM, Alizadeh M and Gutierrez PL (1985) Mechanism for the reductive activation of diazaquinone. *J Pharm Sci* **74**: 1249–1254

Newling D (1990) Intravesical therapy in the management of superficial transitional cell carcinoma of the bladder: the experience of the EORTC GU group. *Br J Cancer* **61**: 497–499

Oosterlink W, Kurth KH, Schröder F, Bultinck J, Hammond B, Sylvester R and members of the European Organisation for Research and Treatment of Cancer Genitourinary Group (1993) A prospective European Organisation for Research and Treatment of Cancer Genitourinary Group randomised trial comparing transurethral resection followed by a single instillation of epirubicin or water in single stage Ta, T1 papillary carcinoma of the bladder. *J Urol* **149**: 749–752

Pan SS, Yu F and Hipsher C (1993) Enzymatic and pH modulation of MMC induced DNA damage in MMC resistant HCT 116 human colon cancer cells. *Mol Pharmacol* **43**: 870–877

Phillips RM (1996) Bioreductive activation of a series of analogues of 5-aziridinyl-3-hydroxymethyl-1-methyl-2-[1H-indole-4,7-dione] prop-β-en-α-ol (EO9) by human DT-diaphorase. *Biochem Pharmacol* **52**: 1711–1718

Phillips RM (1999) Inhibition of DT-diaphorase (NAD(P)H:quinone oxidoreductase, EC 1.6.99.2) by 5,6-dimethylxanthenone-4-acetic acid (DMXAA) and flavone-8-acetic acid (FAA): Implications for bioreductive drug development. *Biochem Pharmacol* **58**: 303–310

Phillips RM, Hulbert PB, Bibby MC, Sleigh NR and Double JA (1992) *In vitro* activity of the novel indolequinone EO-9 and the influence of pH on cytotoxicity. *Br J Cancer* **65**: 359–364

Phillips RM, Loadman PM and Cronin BP (1998) Evaluation of a novel *in vitro* assay for assessing drug penetration into avascular regions of tumours. *Br J Cancer* **77**: 2112–2119

Plumb JA and Workman P (1994) Unusually marked hypoxic sensitisation to indolequinone EO9 and MMC in a human colon tumour cell line that lacks NQO1 activity. *Int J Cancer* **56**: 134–139

Robertson N, Haigh A, Adams GE and Stratford IJ (1994) Factors affecting sensitivity to EO9 in rodent and human tumour cells *in vitro*: DT-diaphorase activity and hypoxia. *Eur J Cancer* **30A**: 1013–1019

Saunders MP, Jaffar M, Patterson AV, Nolan J, Naylor MA, Phillips RM, Harris AL and Stratford IJ (2000) The relative importance of NADPH:cytochrome c (P450) reductase for determining the sensitivity of human tumour cells to the indoloquinone EO9 and related analogues lacking functionality at the C-2 and C-3 positions. *Biochem Pharmacol* **59**: 993–996

Schellens JHM, Planting AST, Van Acker BAC, Loos WJ, De Boer-Dennert M, Van Der Burg MEL, Koier I, Krediet RT, Stoter G and Verweij J (1994) Phase I and pharmacologic study of the novel indolequinone bioreductive alkylating cytotoxic drug EO9. *J Natl Cancer Inst* **86**: 906–912

Schlager JJ and Powis G (1988) MMC is not metabolised by but is an inhibitor of human kidney NAD(P)H:(quinone acceptor) oxidoreductase. *Cancer Chemother Pharmacol* **22**: 126–130

Siegel D, Gibson NW, Preusch PC and Ross D (1990) Metabolism of MMC by DT-diaphorase. Role in MMC induced DNA damage and cytotoxicity in human colon carcinoma cells. *Cancer Res* **50**: 7483–7489

Siegel D, Beall HD, Kasai M, Gibson NW and Ross D (1993) PH dependent inactivation of DT-diaphorase by MMC and porfiromycin. *Mol Pharmacol* **44**: 1128–1134

Siegel D, Franklin WA and Ross D (1998) Immunohistochemical detection of NAD(P)H:Quinone oxidoreductase in human lung and lung tumours. *Clin Cancer Res* **4**: 2065–2070

Smitkamp-Wilms E, Peters GJ, Pinedo HM, Van Arkotte J and Giaccone G (1994) Chemosensitivity to the indoloquinone EO9 is correlated with DT-diaphorase activity and gene expression. *Biochem Pharmacol* **47**: 1325–1332

Smitkamp-Wilms E, Giaccone G, Pinedo HM, Van Der Laan BFAM and Peters GJ (1995) DT-diaphorase activity in normal and neoplastic human tissues: An indicator of sensitivity to bioreductive agents? *Br J Cancer* **72**: 917–921

Tolley DA, Parmar MKB, Grigor KM, Lallemand G and the Medical Research Council superficial bladder cancer working party (1996) The effect of intravesical MMC on recurrence of newly diagnosed superficial bladder cancer: A further report with 7 years of followup. *J Urol* **155**: 1233–1238

Traver RD, Horikoshi T, Dannenberg KD, Stadlbauer THW, Dannenberg PV, Ross D and Gibson NW (1992) NAD(P)H:quinone oxidoreductase gene expression in human colon carcinoma cells: Characterisation of a mutation which modulates DT-diaphorase activity and mitomycin sensitivity. *Cancer Res* **52**: 797–802

Walton MI, Smith PJ and Workman P (1991) The role of NAD(P)H:quinone reductase (EC 1.6.99.2, DT-diaphorase) in the reductive bioactivation of the novel indoloquinone antitumour agent EO9. *Cancer Commun* **3**: 199–206

Workman P (1994) Enzyme directed bioreductive drug development revisited: A commentary on recent progress and future prospects with emphasis on quinone anticancer drugs and quinone metabolising enzymes, particularly NQO1. *Oncol Res* **6**: 461–475

Yen WC, Schmittgen T and Au JL (1996) Different pH dependency of mitomycin C activity in monolayer and three dimensional cultures. *Pharmaceut Res* **13**: 1887–1891

*© 2001 Cancer Research Campaign*
